# A comprehensive phenotypic and genomic characterization of Ethiopian sorghum germplasm defines core collection and reveals rich genetic potential in adaptive traits

**DOI:** 10.1002/tpg2.20055

**Published:** 2020-09-17

**Authors:** Gezahegn Girma, Habte Nida, Alemu Tirfessa, Dagnachew Lule, Tamirat Bejiga, Amare Seyoum, Moges Mekonen, Amare Nega, Kebede Dessalegn, Chemeda Birhanu, Alemnesh Bekele, Adane Gebreyohannes, Getachew Ayana, Tesfaye Tesso, Gebisa Ejeta, Tesfaye Mengiste

**Affiliations:** ^1^ Department of Botany and Plant Pathology Purdue University West Lafayette IN 47907 USA; ^2^ Ethiopian Institute of Agricultural Research P.O. Box 2003 Addis Ababa Ethiopia; ^3^ Oromia Agricultural Research Institute P.O. Box 81265 Addis Ababa Ethiopia; ^4^ Haramaya University P.O. Box 138 Dire Dawa Ethiopia; ^5^ Department of Agronomy Kansas State University 3007 Throckmorton PSC 1712 Claflin Road Manhattan KS 66506 USA; ^6^ Department of Agronomy Purdue University West Lafayette IN 47907 USA

## Abstract

Understanding population genetic structure and diversity of a crop is essential in designing selection strategies in plant breeding. About 2010 Ethiopian sorghum accessions were phenotyped for different traits at multiple locations. A subset of the collection, 1628 accessions, predominantly landraces, some improved varieties, and inbred lines were genotyped by sequencing. Phenotypic data revealed association of important traits with different sorghum growing agro‐climatic regions, high genetic diversity and the presence of rare natural variation in the Ethiopian sorghum germplasm. Subsequent genotypic analysis determined optimum number of sub‐populations, distinct cluster groups and ancestries of each sorghum accessions. To improve utilization of germplasm, a core subset of 387 lines were selected following posteriori grouping of genotypes based on cluster groups obtained through GBS analysis followed by stratified random sampling using quantitative traits. In order to evaluate how well this new sorghum and millet innovation lab (SMIL) collection from Ethiopia is represented within the largest world sorghum collection at United States Department of Agriculture ‐ National Plant Germplasm System (USDA‐NPGS) and the sorghum association panel (SAP), comparisons were conducted based on SNP data. The SMIL collection displayed high genetic diversity with some redundancy with the USDA‐NPGS germplasm but SAP showed clear distinction. Furthermore, genome‐environment association analysis identified candidate genes associated with adaptation to abiotic factors, that will be important for exploitation of adaptive potential to different environments. In summary, our results described the diversity and relationship of sorghum collections, representativeness of developed core and provide novel insights into candidate genes associated to abiotic stress tolerance.

**Abbreviations**
BLINK: bayesian‐information and linkage‐disequilibrium iteratively nested keyway
FDR: false discovery rate
GBS: genotyping‐by‐Sequencing
GEA: genome‐environment association analysis
GWAS: genome‐wide association studies
IBS: identity‐by‐state
LD: linkage disequilibrium
NPGS: national plant germplasm system
PCA: principal component analysis
Q–Q: quantile‐quantile plot
SAP: sorghum association panel
SMIL: sorghum and millet innovation lab
SNP: single nucleotide polymorphism.

## 1 INTRODUCTION

*Sorghum bicolor* (L.) Moench is a staple food crop with particular importance to subsistence farming in semi‐arid tropical regions of the world (Leff, Ramankutty, & Foley, 2004). These areas are known for erratic rainfall and moisture deficit, often not suitable for other cereal crops. Drought tolerance of sorghum is partly attributed to its C4 physiology, a physiological mechanism that makes plants more efficient while energy making in hot, sunny and dry climates, and in turn, results in higher productivity. The C4 photosynthetic carbon cycle has evolved as an adaptation to high light intensities, high temperatures, and drought, which allows them to dominate grassland floras and biomass production in the warmer climates of the tropical and subtropical regions (Edwards, Osborne, Strömberg, & Smith, 2010). Several other adaptive mechanisms such as thick leaf wax, deep root system and stay green trait allow its tolerance under extreme drought condition (Tari, Laskay, Takacs, & Poor, 2013).

Evolved in one of the major centers of crop diversity in the world with wide ranging environmental conditions (Plant Genetic Resources Center, 1995), Ethiopian sorghums harbor significant diversity for important traits such as cold (Singh, 1985), and drought tolerance (Adugna, 2014), disease resistance (grain mold and various leaf diseases) (Nida et al., 2019; Weerasooriya et al., 2016) and nutritional profile (Rhodes et al., 2017). Of the known main sorghum races, i.e. bicolor, guinea, caudatum, dura and kafir, all but kafir are grown in Ethiopia (Engels & Hawkes, 1991). As a result, today's sorghum crop possesses broad ecological adaptation where it is very commonly grown in diverse climates, from the dry lowland areas of the semi‐arid tropics receiving very little annual precipitation, to high rainfall and humid regions, as well as in the more temperate regions of the world.

According to data regularly compiled by the Food and Agricultural Organization (FAO) of the United Nations, from 1994 to 2018, of the top ten sorghum producing nations, more than 67% of the global sorghum production is contributed by developing countries (FAOSTAT, 2020). However, sorghum productivity in developing countries in general and sub‐Saharan Africa in particular, remains low as its production is pre‐dominantly with seeds of low yielding landrace germplasm cultivated in soils that are inherently nutrient deficient, and in regions with erratic rainfall and low/high temperature stresses. Use of modern technologies, inorganic fertilizer and improved seed of sorghum varieties among small holder farmers of this region continues to be low (Mundia, Secchi, Akamani, & Wang, 2019). Moreover, pest and disease limit sorghum productivity (Kumari et al., 2016; Wortmann et al., 2009).

Broad genetic diversity is essential for providing the gene pool necessary for the development and production of new seed varieties of crops with badly needed agriculturally important traits. Unfortunately, vast variation of gene pools is often harbored in raw landrace germplasm or their wild and related species of native crops that evolved in Vavilov centers of origin and diversity of crop plants such as Ethiopia (Hummer & Hancock, 2015). Hence, both the collection and assembly, as well as the phenotypic and genotypic characterization of such a collection, are key steps in a well thought out and careful crop improvement and utilization program. Large‐scale germplasm characterization with next‐generation sequencing based genotyping techniques such as genotyping‐by‐sequencing (GBS), facilitate exploration and discovery of novel traits to expedite genetic gain during crop improvement process by enabling selection of a diverse genetic pool. Several efforts have been made to characterize Ethiopian sorghum germplasm collection ranging from 80 to 974 accessions (Adugna, 2014; Ayana & Bekele, 2000; Ayana, Bryngelsson, & Bekele, 2000; Desmae, Jordan, & Godwin, 2016; Weerasooriya et al., 2016). However, these studies were limited in sample size and lacked representation of the large geographic coverage of Ethiopian sorghum collections that are held at several sites. The Ethiopian Biodiversity Institute (EBI) holds a total of 9249 sorghum accessions (IBC, 2007). A collection of Ethiopian sorghums is also maintained at different genebanks around the world, including over 2000 accessions conserved at the International Crops Research Institute for the Semi‐Arid Tropics (ICRISAT) (Upadhyaya, Sharma, Dwivedi, & Singh, 2014) and about 7400 accessions preserved at genebanks in the National Plant Germplasm System (NPGS) of the United States Department of Agriculture (USDA) (Cuevas, Rosa‐Valentin, Hayes, Rooney, & Hoffmann, 2017). The Ethiopian sorghum germplasm is also represented in the assembly of sorghum association panel (SAP), which mainly includes germplasm that have gone through a conversion program which converted tall, late and photoperiod sensitive tropical sorghum landraces to short, early and photoperiod insensitive lines (Stephens, Miller, & Rosenow, 1967).

Genetic characterization and evaluation of germplasm will improve their utilization in crop improvement programs. Phenotypic characterization and evaluation of germplasm is a requisite step for targeted genomic and genetic resource characterization and utilization in crop breeding programs. However, effective phenotypic assessment and characterization of large genetic populations and/or germplasm collections is generally challenging, as it limits replicated multi‐location characterization with significant cost, and sometimes near‐impossible logistic implications to generating detailed information on many traits of interest. To overcome challenges in effective use of large germplasm collection and facilitate easy access, the concept of core collection was introduced (Frankel, 1984). Core subsets usually represent about 10% of an entire collection and capture the maximum diversity of base collection. The concept and development of core collections has become an important tool in efficient germplasm management, evaluation and utilization (Dahlberg, Burke, & Rosenow, 2004; Yan et al., 2007)

The ever decreasing cost and high throughput sequencing allowed genotyping of many individuals, increasing the power of genome‐wide association studies (GWAS). GWAS have enabled identification of loci and/or genes underlying complex traits, such as yield (Li et al., 2019), disease resistance (Wang et al., 2012) and other quantitative traits (Girma et al., 2019). It is known that quantitative traits are highly influenced by environment and genetics. Genome‐environment association (GEA) studies based on GWAS models have created additional avenue for exploring the genetic basis of plant adaptation to abiotic stresses (Lopez‐Hernandez & Cortes, 2020; Torre, Wilhite, & Neale, 2019).

In this study, phenotypic data from field trials, genotypic data generated through genotyping by sequencing, coupled with agro‐climatic data of sorghum landraces representing Ethiopian sorghum germplasm collection were used to conduct comprehensive germplasm analyses. A representative core subset that captures the diversity of the Ethiopian sorghum germplasm was developed as a critical resource for sorghum breeding, genomic and genetic studies. Furthermore, we implemented genome‐environment association (GEA) studies to explore the genetic basis of adaptive traits. Overall, we provide a better insight into the Ethiopian sorghum germplasm, which is important in the world sorghum collection for its features relative to other collections.

**Core Ideas**

- Ethiopian sorghum germplasm harbors considerable diversity and rare natural variation.
- A representative core subset was developed that will facilitate access to and efficient utilization of germplasm.
- Genetic uniformity arising from recycling of parental sources in breeding programs is a bottleneck that can be overcome by genomic characterization of germplasm.
- A linkage disequilibrium decay pattern (*r* ^2^ = 0.2) within 9 kb across the large sorghum landrace collection improves GWAS efficiency.
- Genome–environment association analyses revealed candidate genes associated with adaptive traits.

## 2 MATERIALS AND METHODS

### 2.1 Plant materials and passport data

A total of about 2010 Ethiopian sorghum germplasm entries comprising landrace, improved varieties and inbred lines were included in this study (Supplemental Table S1). These germplasms were assembled from several sources by the sorghum research project at Ethiopian Institute of Agricultural Research (EIAR), as part of a United States Agency for International Development (USAID) funded sorghum and millet innovation lab (SMIL) project, and the collection is hereafter described as the SMIL collection. Most of the accessions representing landraces were derived from a larger collection of sorghum germplasm maintained at Ethiopian Biodiversity Institute (EBI). Representative samples were drawn from a list of more than 9000 accessions by taking major sorghum geographic regions, occurrence of different stressors, local production, and utilization practices into consideration. The original passport data included only accession and locality names. Passport data was further enriched by including mean annual temperature (°C), annual precipitation (mm), altitude (m asl) and agro‐climatic information following commonly used agro‐climatic and Köppen‐Geiger climate classification (Kottek, Jurgen, Christoph, Bruno, & Franz, 2006) for each accession based on available locality name (Supplemental Table S1). The traditional agro‐climatic classification is largely based on mean annual precipitation and altitude.

### 2.2 Test sites and phenotyping

All accessions (*N* = 2010) were planted at Bako (9°08′ N latitude, 37°03′ E longitude, 1642 m asl altitude, 19.7 °C mean annual temperature, 1281 mm mean annual rainfall), Arsi Negelle (7°21′ N latitude, 38°42′ E longitude, 1951 m asl altitude, 17.7 °C mean annual temperature, 915 mm mean annual rainfall) and Haramaya (9°24′ N latitude 42°01′ E longitude, 2047 m asl altitude, 17.9 °C mean annual temperature, 799 mm mean annual rainfall). Each accession was planted in a single row of 3 m length with 20 cm distance between plants and 80 cm between plots during beginning of May to mid‐May of 2015 and 2016 at Arsi Negelle, Haramaya and Bako. Fertilizer was applied at the rate of 100 kg/ha DAP at planting and 50 kg/ha urea at knee height. Pure lines in each accession were obtained by selecting and selfing five single heads representing the most frequent genotype within a row. The homogeneity was confirmed by planting individuals for at least one more season. Five plants representing each accession were tagged and data on sixteen morphological traits (Supplemental Table S2) were collected following the standard descriptors (IBPGR and ICRISAT, 1993) used in sorghum germplasm characterization. Six quantitative traits (days to flowering, days to physiological maturity, plant height, seed number, grain yield and thousand kernel weight) were collected from the three locations. SPAD and all categorical traits were collected at Arsi Negelle (Supplemental Table S2).

### 2.3 Genotyping‐by‐sequencing

A total of 1628 accessions including 1585 landrace, 32 cultivars and 11 inbred lines were genotyped by sequencing (Supplemental Table S1). Of which 40 individuals were genotyped redundantly as intentional duplicates to determine any genotyping error and exclude identical accessions; where 37 were in duplicate, two in triplicate and one seventeen times. DNA from dried tissue of week‐old seedlings after germination was isolated following a modified CTAB procedure (Mace, Buhariwalla, Buhariwalla, & Crouch, 2003). A total of seventeen 96‐plex ApeKI reduced representation libraries were constructed and genotyped following genotyping by sequencing (GBS) procedure (Elshire et al., 2011) at University of Wisconsin, Biotechnology Center. SNP datasets are deposited in Purdue University Research Repository https://purr.purdue.edu/publications/3189/2 (https://doi.org/10.4231/EZDX-XT41).

In addition, other datasets (Supplemental Table S3) previously generated through similar genotyping procedure were included with the current SMIL dataset for further analysis. The materials represent 285 SAP lines among genotypes used in previous studies (Morris et al., 2013) and a core subset of 380 accessions representing 5% of the 7400 Ethiopian sorghum germplasms maintained at USDA‐NPGS as part of the largest global sorghum collection previously described (Cuevas et al., 2017).

### 2.4 Data analysis

#### 2.4.1 Phenotypic data analysis

Descriptive statistics (range, mean and standard error) were computed for seven quantitative traits by categorizing accessions into different agro‐climatic groups, commonly used classification of sorghum growing areas in Ethiopia. Pearson correlation coefficients and confidence interval of the correlation coefficient at 95% were computed across quantitative traits using mean values from all the locations. However, as SPAD data was collected only at Arsi Negelle its correlation coefficient was also computed with other quantitative traits obtained from the same site. Frequency distribution for categorical data across all accessions were visualized using histogram.

#### 2.4.2 GBS variant calling and quality control

Single nucleotide polymorphism (SNP) calling was performed using the *DiscoverySNPCallerPluginV2* of the TASSEL GBS pipeline v5 (Glaubitz et al., 2014) following sequence read alignment to the reference genome of *Sorghum bicolor* version 3.1.1 (McCormick et al., 2018) using Burrows ‐Wheeler Alignment (BWA) tool (Li & Durbin, 2009). The SNP calling analysis was done for SMIL collection separately, and in unison with other datasets representing SAP lines and Ethiopian sorghum core subset at USDA‐NPGS. Quality control of SNP data was performed considering percent missing individual, missing marker, minor allele frequency and excess heterozygosity with PLINK version 1.90 (Purcell et al., 2007).

#### 2.4.3 Population genetic structure and genetic diversity analysis

Pairwise genetic distance was calculated among accessions genotyped in duplicate. Any variation between two individuals from the same genotype was considered due to genotyping error. Accordingly, genetic distance threshold was set for declaring two accessions as identical. To describe population structure of the SMIL collection, different complementary approaches were implemented. Hierarchical clustering and neighbor joining tree were made by calculating pairwise genetic distance (identity‐by‐state, IBS) in PLINK version 1.90 (Purcell et al., 2007). A Ward's minimum variance hierarchical cluster dendrogram was then built from IBS matrix using an R (R Core Team, 2017) package called Analyses of Phylogenetics and Evolution (APE) (Paradis, Claude, & Strimmer, 2004). Secondly, model‐based maximum likelihood estimation of individual ancestries from multi‐locus SNP genotype datasets using ADMIXTURE method (Alexander & Lange, 2011) which assumes linkage equilibrium among loci and Hardy‐Weinberg equilibrium within ancestral populations was used to identify ancestries of each sorghum accession. The admixture analysis was performed for different K (number of sub‐populations) varying from 2 to 20. The most appropriate K‐value was selected after considering 10‐fold cross‐validations whereby the best K exhibits low cross‐validation error compared to other K values.

To further understand patterns of genetic relatedness across accessions, principal components analysis (PCA) was conducted using ‘prcomp’ function in R (R Core Team, 2017). This was further looked in terms of partitioning of genomic variation due to improvement status (landrace/released landrace/improved variety/inbred line) and germplasm source (SMIL/USDA‐NPGS/SAP) across sorghum accessions. Genetic diversity parameters which include inbreeding coefficient (FIS), observed heterozygosity (Ho), expected heterozygosity (He), minor allele frequency (MAF) and polymorphic information content (PIC) across sorghum accessions were generated using TASSEL (Glaubitz et al., 2014) and PLINK version 1.90 (Purcell et al., 2007).

#### 2.4.4 Linkage disequilibrium (LD) analysis

Linkage disequilibrium (LD) which is described as the non‐random association between alleles at different loci is an important parameter in determining resolution of association mapping studies. In this study we generated pairwise LD for all SNPs reported based on adjacent pairwise *r^2^* values across the genome using a command line in PLINK (Purcell et al., 2007). LD decay with genetic distances in kb between pairs of loci was generated using ggplot2 package (Wickham, 2016) in R (R Core Team, 2017) for populations representing the three collections by taking mean values from 10 chromosomes.

#### 2.4.5 Core subset selection and validation

To further improve utilization of germplasm, a core subset was selected following posteriori grouping of genotypes (1941 accessions representing landrace) based on genetic cluster group obtained through GBS analysis followed by stratified random sampling using quantitative traits. The optimum number of K with lower cross validation error value determined through admixture analysis was considered as the most appropriate number of sub‐populations. All genotyped accessions were classified into one of the sub‐populations and remaining accessions with no genotype data were treated separately as an independent group. Stratified selection of core subset as previously suggested (Franco, Crossa, Taba, & Shands, 2005) was made using a ccChooser package (Studnicki & Debski, 2012) in R software (R Core Team, 2017). The core selection was validated by comparing differences in range, mean and variances of entire and core collections for quantitative traits. Shannon‐Weaver diversity index (H) (Shannon, C. E. & Weaver, 1949) was used to check frequency distribution for categorical traits. Chi‐square (χ^2^) probability was used to determine frequency distribution based on agro‐climatic group. Furthermore, neighbor joining tree for all genotyped landrace accessions was generated to visualize distribution of the core selected across entire collection.

#### 2.4.6 Genome‐environment association (GEA) analysis

Agro‐climatic information including altitude, mean annual temperature and precipitation for each locality, representing the collection area of 1425 sorghum landrace accessions and robust SNP markers that passed through quality control from GBS data were used. An association analysis was performed with various GWAS models including general linear model (GLM) with genotype data corrected for population structure, mixed linear model (MLM) with genotype data corrected for both population structure and kinship (Zhang et al., 2010) and Bayesian‐information and linkage‐disequilibrium iteratively nested keyway (BLINK), an enhanced version of the fixed and random model circulating probability unification (FarmCPU) method (Huang et al., 2018), that substitutes kinship with a set of markers associated with the casual genes. GWAS was implemented in the GAPIT package (Lipka et al., 2012) with the three different models in R software (R Core Team, 2017). Log Q–Q plots of p‐values were examined to determine how well the models accounted for population structure. Significant associations were determined for each trait by adjusting the raw p‐value using the conservative Bonferroni error rate control method. Manhattan and Q–Q plots were visualized using the qqman R package (Turner, 2014). All the significant SNP markers were mapped onto the most recent version of *Sorghum bicolor* v3.1.1 (McCormick et al., 2018) genome in Phytozome v12.1 (Goodstein et al., 2012).

## 3 RESULTS

### 3.1 Geographic description of the study materials

**FIGURE 1:**
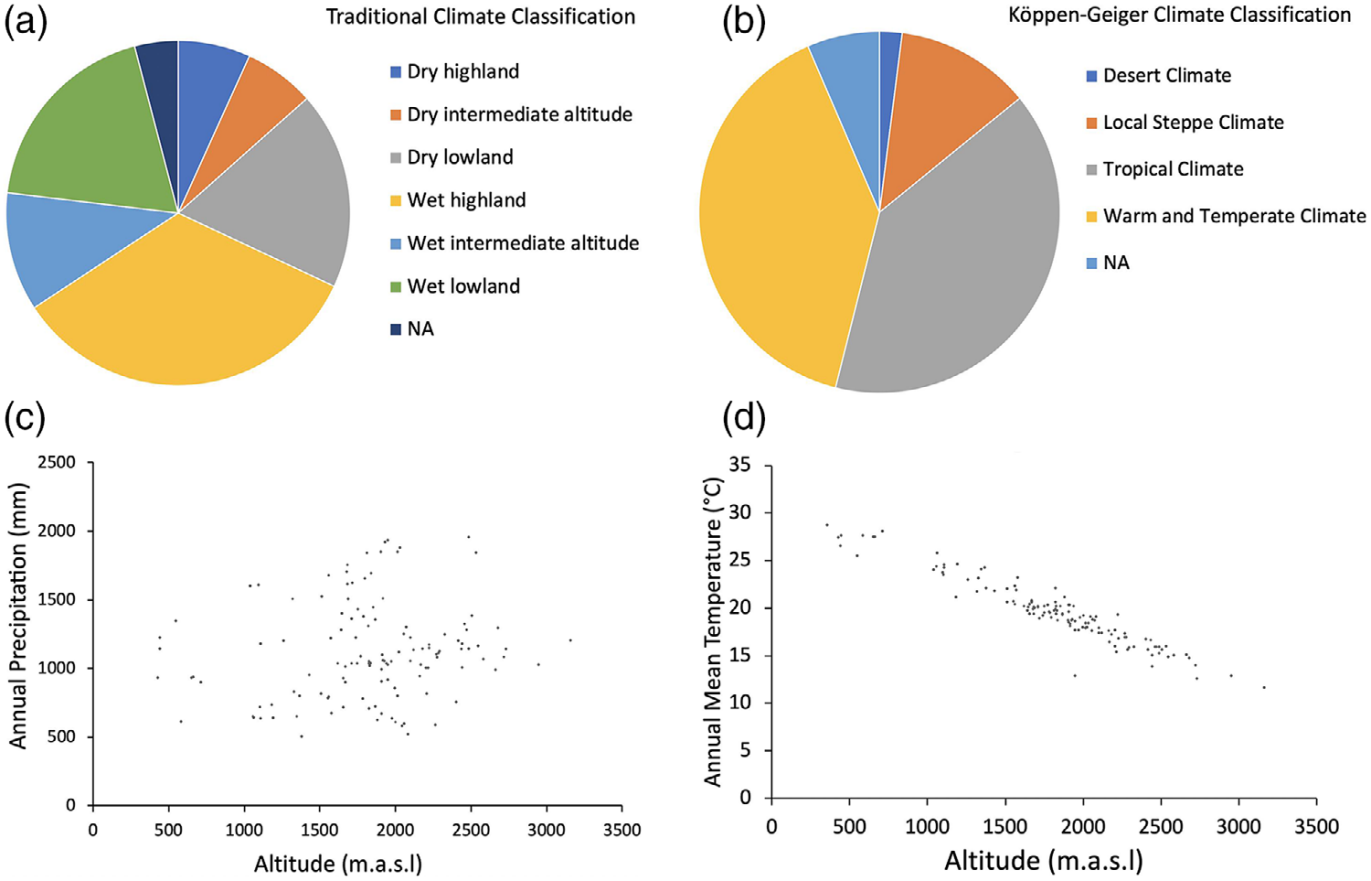
Agro‐climatic classification of Ethiopian sorghum accessions in the SMIL collection. (a) traditional climate classification, (b) Köppen‐Geiger climate classification, (c) annual mean precipitation and (d) annual mean temperature across a range of altitudes of sorghum growing areas. NA represents number of accessions that could not be assigned to any of the agro‐climatic groups due to missing information

The current collection was the largest studied both in terms of sample size and representation across sorghum growing agro‐ecologies of Ethiopia; local steppe climate to warm and temperate climate with elevation ranging from 356–3162 m asl, mean annual temperature from 11.6–28.7 °C and mean annual precipitation from 144–1955 mm (Figure 1; Supplemental Table S1). Accessions from highland areas with altitude above 1900 m asl represented the largest collection followed by dry lowland, wet lowland and intermediate altitudes. Likewise, grouping according to Köppen‐Geiger climate classification indicated large number of accessions from tropical climate followed by warm and temperate climate with the least number of accessions from local steppe climate. The weather data also showed insignificant but a positive correlation (*r* = 0.06) between mean annual precipitation and altitude. Whereas, strong negative correlation (*r* = −0.96) were observed between mean annual temperature and altitude.

### 3.2 Phenotypic diversity across SMIL collection

**FIGURE 2:**
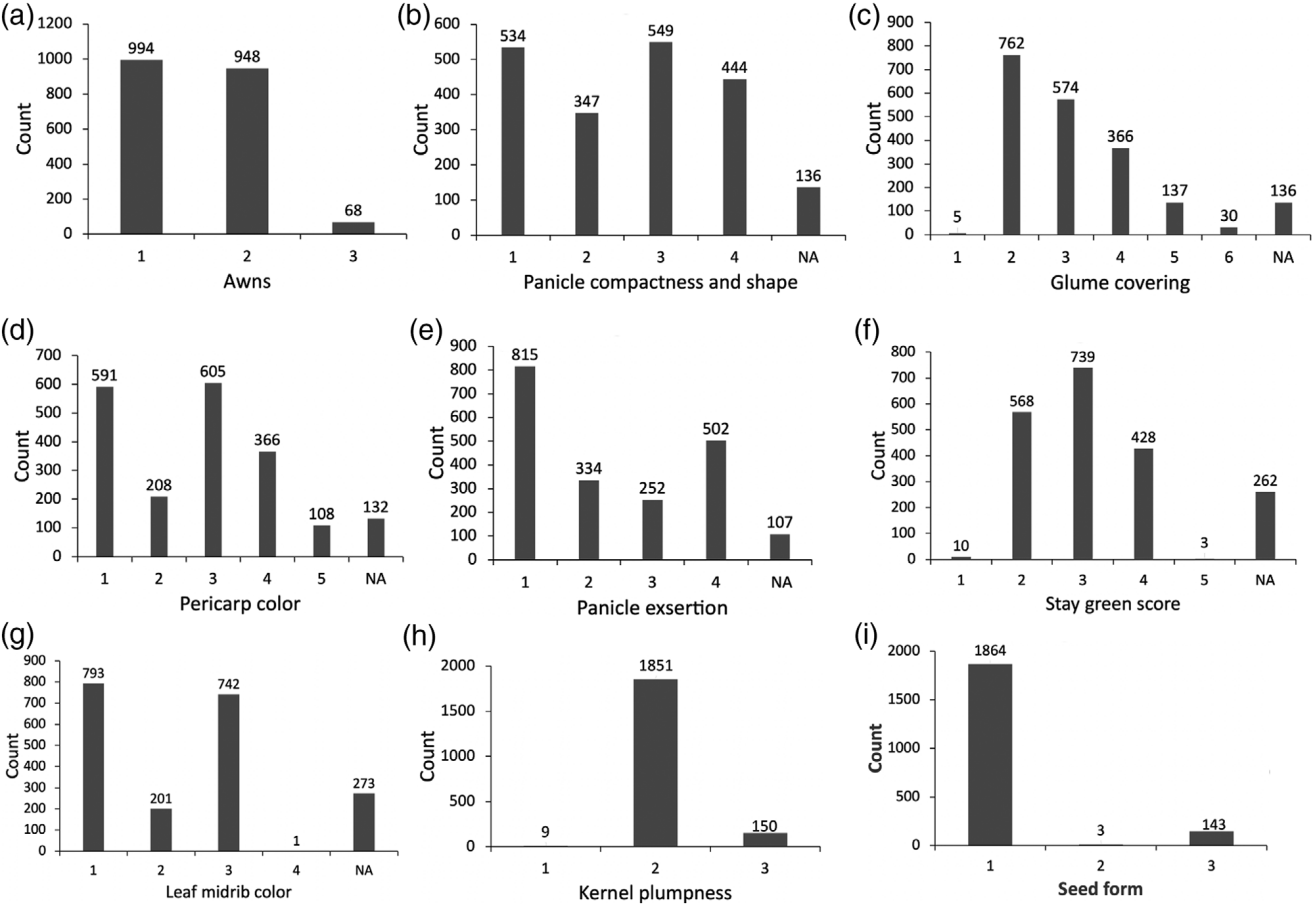
Summary on categorical phenotypic traits across 2010 sorghum accessions. (a) awns (1 = awned and 2 = awnless), (b) panicle compactness and shape (1 = loose erect, 2 = loose drop, 3 = compact elliptic (erect)) and 4 = compact oval or recurved), (c) glume covering (1 = grain uncovered, 2 = 25% of grain covered, 3 = 50% of grain covered, 4 = 75% of grain covered, 5 = grain fully covered, 6 = glumes longer than grain), (d) pericarp color (1 = white, 2 = yellow, 3 = red, 4 = brown and 5 = buff), (e) panicle exsertion (1 = panicle well exserted with 10 cm between ligule of flag leaf to panicle base, 2 = 2–10 cm exsertion, 3 = less than 2 cm but ligule below the panicle base, 4 = peduncle recurved but panicle is below the ligule and clearly exposed splitting the leaf sheath), (f) stay green (1 = more green and 5 = less green), (g) leaf midrib color (1 = colorless (white), 2 = dull green, 3 = yellow and 4 = brown), (h) kernel plumpness (1 = dimple and 2 = plump) and (i) seed form (1 = single and 2 = twin, 3 = unidentified)

Ethiopian sorghum germplasm, currently studied under SMIL collection, represents diverse set of materials based on both quantitative and qualitative traits. Qualitative data collected in the study were summarized and presented in histograms (Figure 2). All quantitative traits measured were grouped and summarized based on agro‐climatic information (Supplemental Table S4). The highest mean plant height of 331.33 cm was observed in germplasm from wet intermediate altitude with shorter plant types from the dry lowland areas. Likewise, both days to flowering and maturity were longer in the wet intermediate altitude accessions and shorter in dry lowland materials. The highest mean number of seeds per panicle was also observed among accessions from wet intermediate altitude with the least number of seeds recorded in dry lowland materials. However, thousand grain weight and the SPAD (soil plant analysis development) values were higher among germplasm from dry areas and lower in materials from wet intermediate altitude. Grain yield per panicle was highest in the dry intermediate altitude accessions and least in wet lowland types. Overall, plant height ranged from a fairly dwarf type of about 100 cm to as tall as 500 cm, flowering from 76 to 157 days, maturity period ranging from 136 to 208 days, thousand grain weight (TGW) from 7 to 52 gm, grain yield per panicle (GY) from 0.1 to 213 gm, seed number from 20 to 8070 per head and SPAD values from 19.70 to about 70. Overall, Pearson correlation analysis among quantitative traits revealed significant association, except for non‐significant and negative association between SPAD score and seed number per panicle (Supplemental Table S5). SPAD also showed significant negative correlation with DTF (*r* = −0.16, *P* ≤ .01), DTM (*r* = −0.39, *P* ≤ .01) and PHT (*r* = −0.45, *P* ≤ .01). However, the correlation between SPAD and TGW (*r* = 0.24, *P* ≤ .01) and GY (*r* = 0.08, *P* ≤ .01) was significant and positive.

### 3.3 Summary statistics of SNP markers

A total of 879,407 SNP markers were initially discovered across 1628 individuals genotyped within SMIL collection. Overall, 46% of the SNPs called in the entire accessions had minor allele frequency (MAF) <0.05. Quality control of SNP data based on individuals with <20% missing and markers with <40% missing and MAF >0.05 produced 72,190 robust SNP markers with 1605 individuals passing filtering criteria. The number of SNPs was the lowest on chromosome 7 and the highest on chromosome 1. Both MAF and polymorphic information content (PIC) were the highest on chromosome 9 and lowest on chromosome 6. The highest and lowest level of heterozygosity was observed on chromosome 5 and chromosome 6, respectively (Supplemental Figure S1a). Overall, high‐density SNP markers were observed toward the tails across all chromosomes with almost no SNPs mostly in centromeric regions (Supplemental Figure S1b).

Variant calling based on combined dataset of 2198 individuals (1541 from SMIL landrace collection, 285 SAP and 380 USDA‐NPGS) resulted in large missing SNP markers across SAP population. This is likely due to improvement in sequence quality over time and batch effect. Hence, we implemented k nearest neighbors’ algorithm in Tassel (Glaubitz et al., 2014) for imputation of missing data. SNP calling for 2198 individuals initially discovered 646,413 SNPs. Quality control of SNP data based on individuals with <20% missing and markers with <60% missing and MAF >0.05 produced 106,330 SNP markers. SNP markers discovered following imputation were used for diversity analysis to compare USDA‐NPGS and SAP lines with SMIL collection.

### 3.4 Population genetic structure across SMIL collection

**FIGURE 3:**
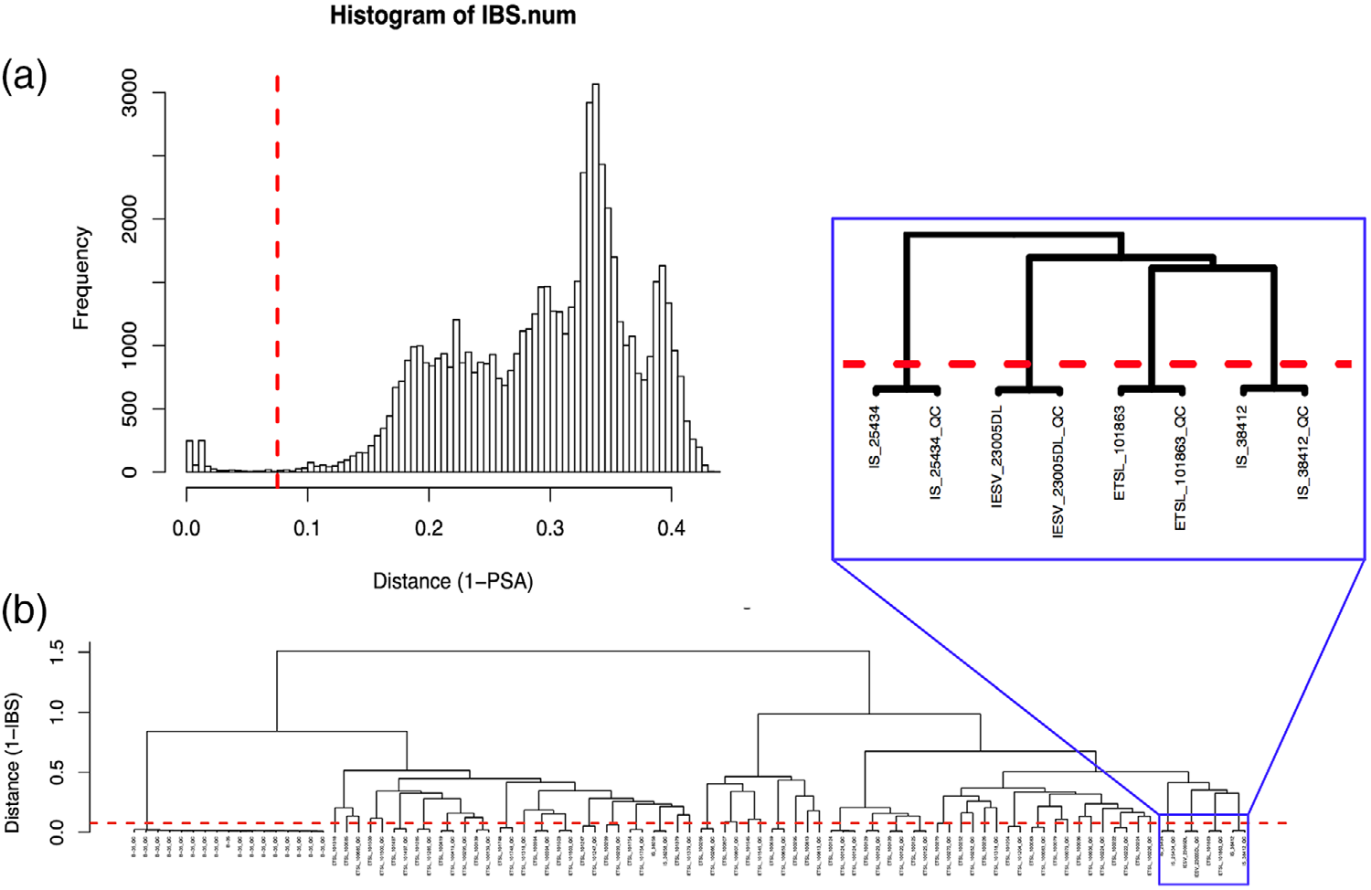
Genetic distance threshold determined for declaring two accessions as identical. (a) bi‐modal distribution of pair‐wise genetic distance across 40 accessions used as intentional duplicates (37 = 2x, 2 = 3x and 1 = 17x) and (b) dendrogram of duplicated DNA samples

**FIGURE 4:**
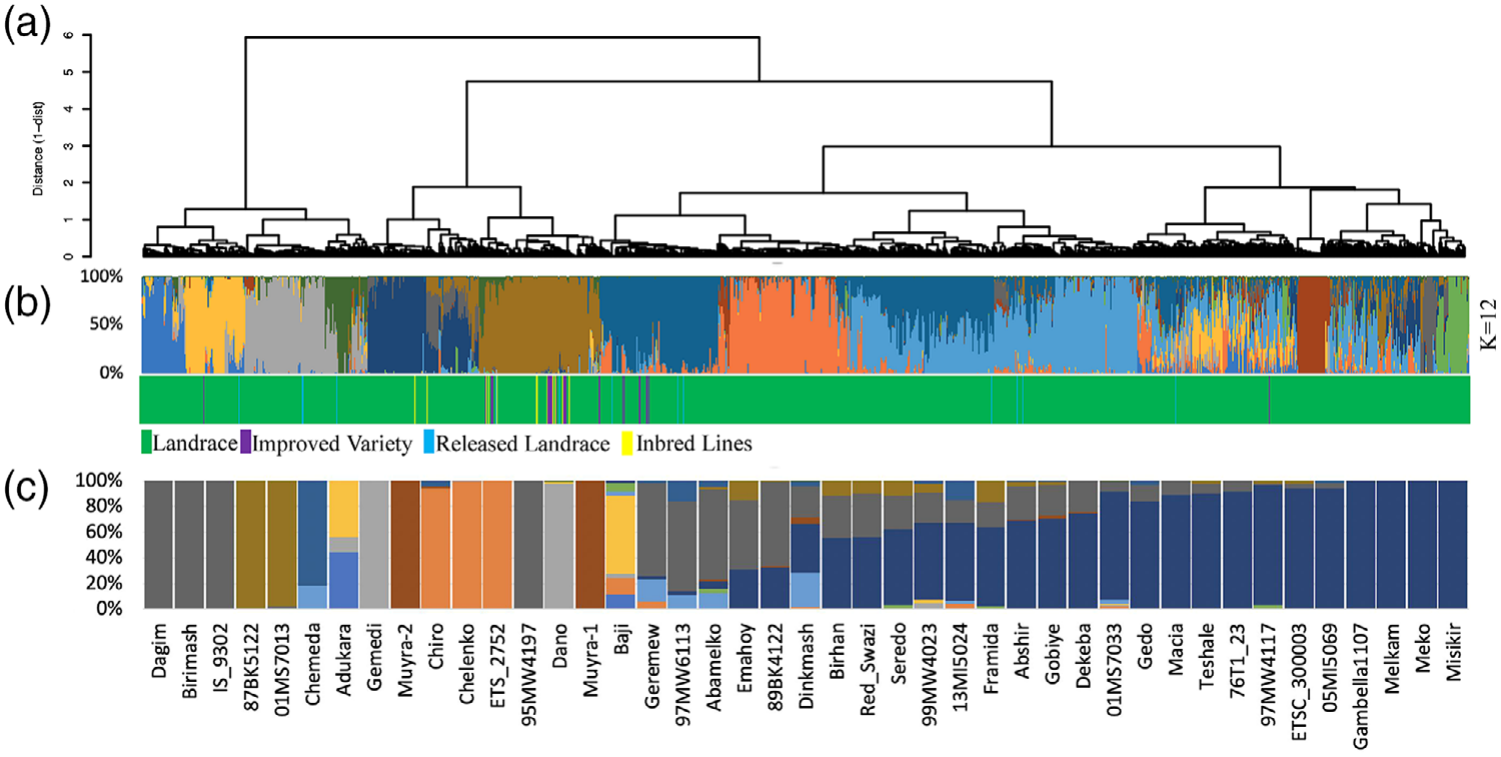
Population genetic structure across SMIL collection. (a) hierarchical clustering (Ward's minimum variance method) dendrogram, (b) individual ancestry estimated based on ADMIXTURE analysis for K = 12. Each individual accession is represented as a single vertical line partitioned into segments corresponding to the inferred membership according to different K genetic clusters as indicated by the colors. The side bar corresponds to improvement status and (c) ancestry fraction estimated based on ADMIXTURE analysis across Ethiopian improved sorghum lines and commonly cultivated landrace at K = 12. The horizontal bar at the side corresponds to improved cultivars (green), inbred lines (yellow) and released landrace (blue)

Following genetic distance analysis among duplicate accessions, a threshold value of 0.075 was established (Figure 3). Subsequently, a total of 1553 genetically distinct accessions (1501 landraces, 32 cultivars, 11 inbred lines and nine genotypes not assigned to any of these groups) were identified and used for further analysis (Supplemental Table S6). Analysis on pairwise distance‐based hierarchical clustering identified distinct cluster groups based on 72,190 SNP markers (Figure 4a). Model‐based maximum likelihood estimation of individual ancestries from multi‐locus SNP genotype datasets using ADMIXTURE identified ancestries of each sorghum accessions. Admixture analysis also revealed K = 12 (Figure 4; Supplemental Figure S2; Supplemental Table S7), as the most appropriate number of sub‐populations and showed good correspondence with the clustering pattern obtained by hierarchical cluster tree. PCA applied to genotype data to compare association between diversity and agroclimatic conditions of collection area explained differences among accessions. The first two principal components (PCs) explained 49.45% of the total variation with accessions from wet highland, wet lowland and wet intermediate altitude showing some tendency to form distinct cluster group (Supplemental Figure S3).

### 3.5 Genetic relationship and ancestry among cultivars and inbred lines

A total of 43 genotypes representing 20 improved cultivars, 11 inbred lines and 12 released landraces were also analyzed for diversity and maximum likelihood estimation of individual ancestries (Figure 4c; Supplemental Table S8; Supplemental Figures S4 and S5). Two groups of improved cultivars including Melkam, Meko and Misikir among the first and Dagim, Birimash and IS9302 in the second group shared more than 99% ancestry within group. Almost all improved cultivars except Baji have backgrounds of mainly these two groups. Baji is a highly admixed cultivar with genetic background from multiple genotypes and significant contribution from released landraces including Adukara and Chiro/Chelenko/ETS2752 group. Interestingly, improved cultivars, Melkam/Meko/Misikir group, shared more than 99% ancestry with a commonly cultivated released landrace, Gambella1107. Released landraces mostly have distinct background but some of them shared high genetic ancestry. Chiro, Chelenko and ETS2752 shared more than 94%; Muyra‐1 and Muyra‐2 shared more than 99% and Dano and Gemedi shared more than 97% ancestry.

### 3.6 Sorghum core subset

A core subset of 387 accessions representing 20% of the SMIL collection at EIAR were selected (Supplemental Table S1). Shannon‐Weaver diversity index across the two groups for categorical traits also confirmed that the core set captured maximum diversity and represented the entire collection (Supplemental Table S9). Frequency comparison based on Chi‐square for entire and core collection showed no significant difference at *P* < .05 between the entire and core collection (Supplemental Table S10). Likewise, core evaluation based on range, mean and variance comparison of the entire and core collection showed no significant differences at *P* < .05 for all the seven morphological traits (Supplemental Table S11). Neighbor joining tree for 1501 genotyped accessions with 72,190 SNPs clearly indicated an even distribution of the core selected lines across all groups (Supplemental Figure S6).

### 3.7 Comparison of genetic diversity, population structure and genome‐wide linkage disequilibrium

To compare and evaluate diversity captured in the SMIL collection, we have included SAP and a core subset representing 5% of 7400 Ethiopian sorghum germplasm within global collection maintained at USDA‐NPGS. The SMIL collection in the current study represented higher diversity with mean genetic distance value of about 0.30 followed by 0.28 in the USDA‐NPGS core collection and the lowest genetic distance of 0.27 was observed in the SAP. Histogram showing genetic distance frequency distribution across genotypes indicated maximum values above 0.43 within SMIL collection and less in the other collections (Supplemental Figure S7). Summary of genetic diversity statistics per taxa showed higher mean proportion of heterozygosity in SMIL followed by USDA‐NPGS core subset. Likewise, per marker statistics revealed high mean PIC, MAF, expected (He) and observed (Ho) heterozygosity among SMIL population, followed by USDA‐NPGS core subset. Conversely, higher mean inbreeding coefficient (F_IS_) were observed in the SAP population with the least in SMIL collection (Supplemental Table S12).

**FIGURE 5:**
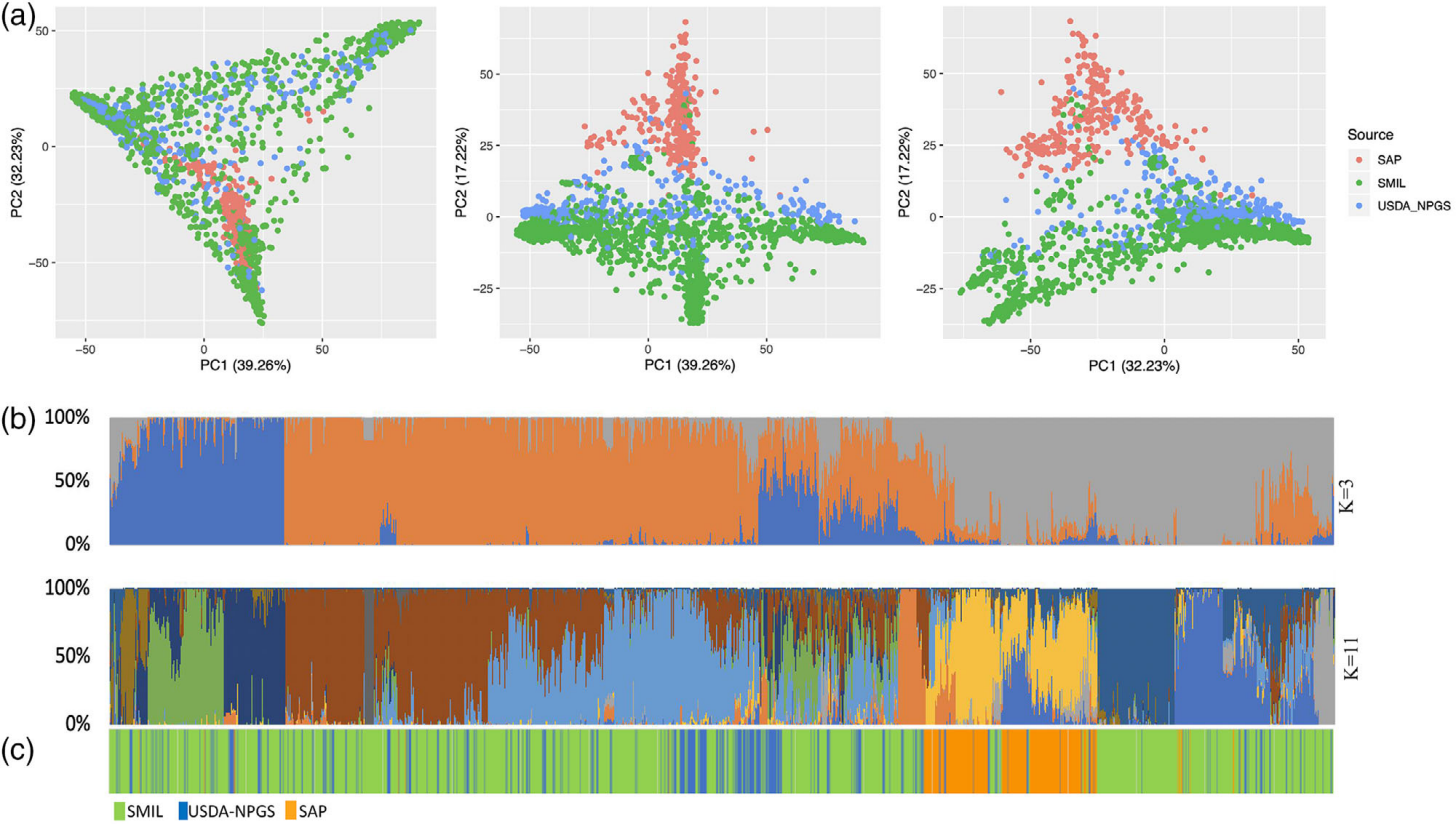
Population genetic structure across sorghum accessions obtained from different source. (a) PCA of 2198 individuals with PC axis calculated using 106,330 SNPs. The proportion of the variance explained by each PC is shown in parentheses. Dots with different color represents SAP (red), SMIL (green) and USDA‐NPGS (blue), (b) individual ancestry estimated based on ADMIXTURE analysis for different K values. Each individual accession is represented as a single vertical line partitioned into segments corresponding to the inferred membership. The lower bar corresponds to germplasm source

Principal component analysis (PCA) clearly revealed distinction of the SAP with the first two principal components explaining 71.49% of the total variation (Figure 5a). The USDA‐NPGS core subset collection showed higher diversity than SAP but was less diverse than the SMIL collection. Model‐based maximum likelihood estimation of individual ancestries from multi‐locus SNP genotype datasets using ADMIXTURE identified ancestries of each sorghum accessions (Figure 5b; Supplemental Table S13). A high ancestry membership coefficient (≥ 0.60) were observed at K = 11 among 68.95% of the genotypes within SMIL, 53.24% within USDA‐NPGS and 47.92% in the SAP population, with remaining respective percentages showing admixed accessions.

**FIGURE 6:**
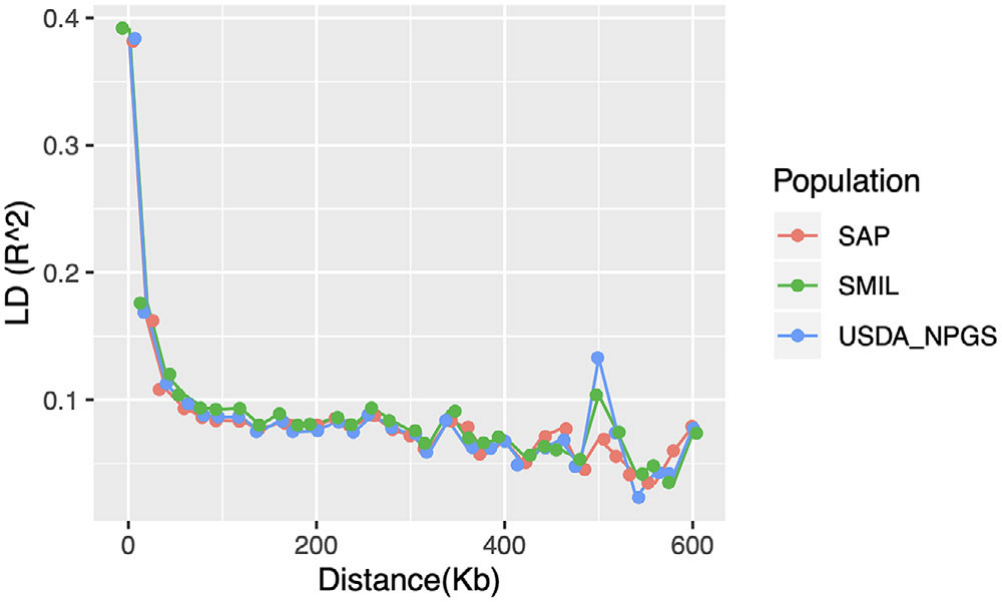
Genome‐wide linkage disequilibrium (LD) decay comparison between SMIL collection, USDA‐NPGS and SAP populations

Genome‐wide LD measured as *r^2^* (the squared correlation coefficients among alleles at two adjacent SNP markers) equals to 0.20 within 6 kb, 7 kb and 9 kb on average in SAP, USDA‐NPGS and SMIL populations, respectively (Figure 6). The LD decays to *r^2^* < 0.1 within 45 kb in the SAP, 55 kb in USDA‐NPGS and 65 kb in the SMIL.

### 3.8 Genome‐environment association study for agro‐climatic parameters

**FIGURE 7:**
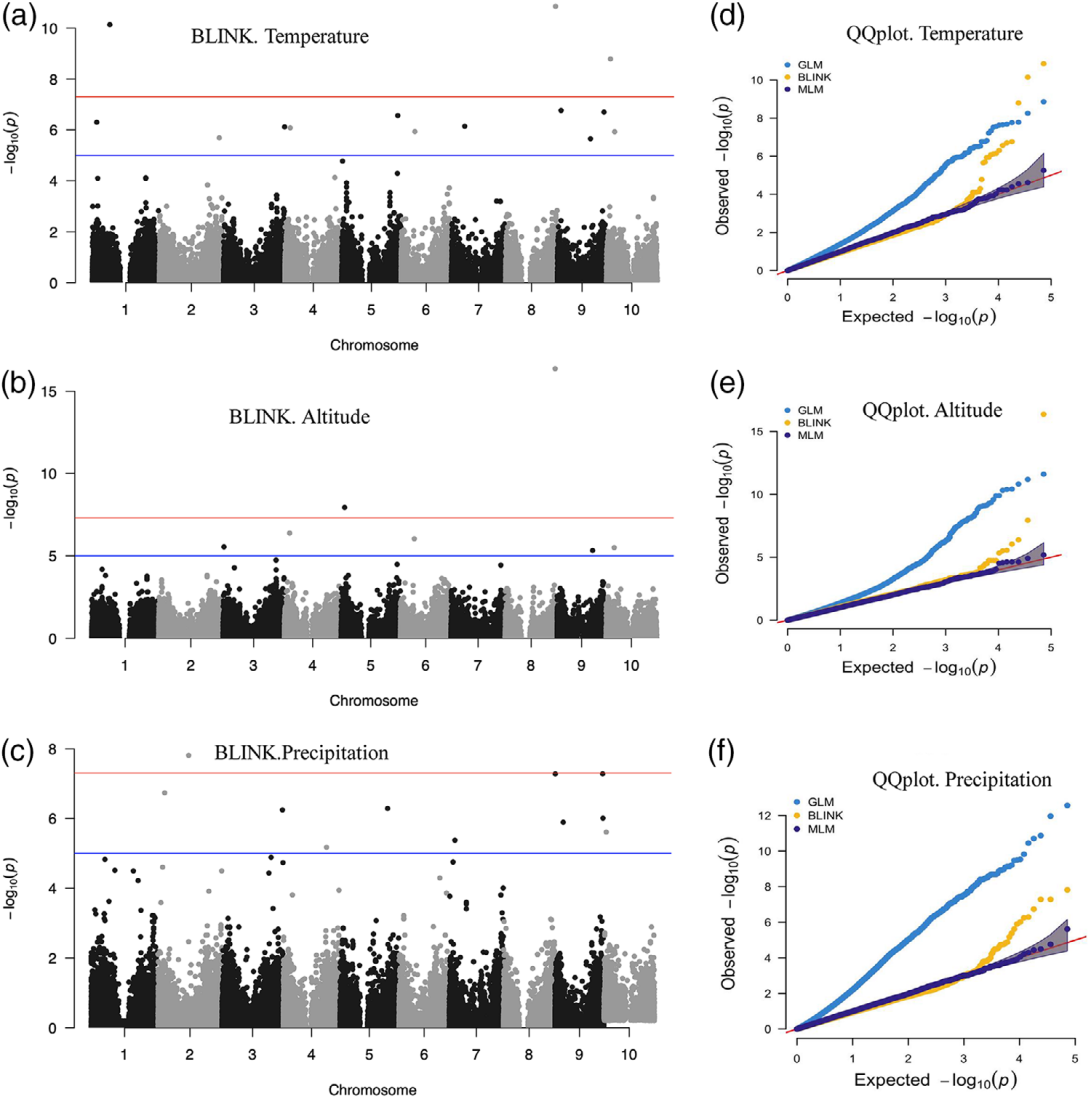
Loci significantly associated with agro‐climatic traits. Manhattan plots of genome‐environment association analysis (a, b and c) and Quantile‐Quantile (QQ) plots with different GWAS models for different agro‐climatic traits (d, e and f). The horizontal red and blue lines represent FDR adjusted *p*‐values < .01 and < .05, respectively

Genome‐environment association (GEA) analysis was conducted using genotype data of 1425 sorghum landraces with their respective agro‐climatic information including mean annual temperature, mean annual precipitation and altitude. The same genotypic data has been used in our recent GWAS analysis across multiple traits which validated previous observations particularly for plant height and pericarp color (Girma et al., 2019). In addition, the distribution of the current agro‐climatic dataset showed approximately normal distribution (Supplemental Figure S8). Three GWAS models were implemented and evaluation of Q‐Q plot across different methods in our GEA analysis indicated BLINK as the most appropriate model, reducing model‐based errors, compared to GLM and MLM (Figure 7d, e and f). GEA results based on BLINK were therefore considered for further candidate gene identification following error rate control of raw p‐values with Bonferroni correction.

A total of 15 different SNPs with significant association, with Bonferroni correction at an error rate of ≤ 0.05, were identified for the three agro‐climatic parameters (Figure 7; Supplemental Table S14). Subsequently, 23 unique candidate genes at or closest to the significant SNPs for all agro‐climatic parameters were identified.

A total of seven SNPs on chromosome 1, 5, 8, 9 and 10 were significantly associated (*P* ≤ 4.27 × 10^−17^) with variation in temperature (Supplemental Table S14). In total, 16 candidate loci at or within 10 kb vicinity of the significant SNPs were identified. Among these, one represents non‐genic region, four were with in coding sequences for genes with no annotation or known protein domains and the remaining 11 represented genes with annotation. Of these, at least five were described to have biological function in plant adaption to temperature variation. The top most significantly associated SNP (S8_60346825), on chromosome 8, was located 2 kb from *Srp40 (Serine/Arginine‐rich proteins*) which was previously reported to contribute to plant‐specific forms of developmental regulation and environmental adaptation (Califice, Baurain, Hanikenne, & Motte, 2012). Four other candidate genes encoding F‐box proteins*, Auxin response factor 22, Pyrophosphate‐energized vacuolar membrane proton pump (EC 3.6.1.1)*, and *Ferritin 2* proteins were reported to mediate auxin action in biotic and abiotic stress responses, regulation of salt and drought stress tolerance and protection against oxidative stress (An et al., 2019; Bouzroud et al., 2018; Briat et al., 2010; Fukuda, Chiba, Maeda, Nakamura, & Maeshima, 2004).

Three SNPs in the vicinity of genic regions were associated with variation in altitude. A single SNP (S8_60346825) closely linked (within 2 kb) to Sobic.008G169200 which encodes a protein weakly similar to suppressor protein *Srp40* and Sobic.008G169000, encoding an *F‐box* protein were significant for plant adaptation to altitudinal variation. In addition*, Serine/Threonine‐protein kinase wnk with no lysine ‐related* which is known to respond differentially under various abiotic stresses including cold, heat, salt and drought (Kumar, Rao, Biswas, & Sinha, 2011) carried SNPs associated with temperature variation. Interestingly, this gene was also reported for its possible role in regulation of flowering time (Wang et al., 2008). Three of the six regions represent uncharacterized proteins with no functional annotation domain.

GEA analysis also identified 13 regions with six SNPs significantly associated (*P* ≤ 1.54 × 10^−8^) to variation in mean annual precipitation. Of which, nine represented transcripts either lacking annotation domain or uncharacterized putative protein. The remaining four candidate regions are annotated to carry a *Myb3 transcription factor*, *Malate dehydrogenase*, *Phosphoribulokinase/Uridine kinase* and small *GTP‐binding* protein, were reported for their roles in adaptation to a various environment (Supplemental Table S14). These genes were previously described to control response to various biotic and abiotic stresses (Agarwal et al., 2008; Ambawat, Sharma, Yadav, & Yadav, 2013; Imran, Tang, & Liu, 2016; Janiak, Kwasniewski, Sowa, & Gajek, 2018).

## 4 DISCUSSION

### 4.1 Ethiopian sorghum genetic diversity and potential implications

Previous reports (Cuevas et al., 2017; Plant Genetic Resources Center, 1995) have described the Ethiopian sorghum as the most diverse crop distributed over a wide range of ecological variation in the country, ranging between 400 and 3000 m asl. The SMIL collection originally obtained from altitude ranges of 356–3162 m asl is, therefore, representative and captured variation in terms of both agro‐climatic and trait variation. All qualitative traits we have described in this particular study have captured all descriptor categories for the traits (IBPGR and ICRISAT, 1993). Plausibly, high agro‐ecological variability and existence of almost all sorghum race types explains the underlying diversity (Engels & Hawkes, 1991).

Of the different sorghum growing agro‐ecological regions, wet intermediate altitude areas represent accessions with taller mean plant height, longer maturity period and high number of seeds per head compared to dry, wet lowland, and highlands (Supplemental Table S4). The wetter regions mostly represent the western parts of the country that is generally fertile, warm with high rainfall which is favorable for sorghum growth. However, the enormous potential of wet lowlands has not yet been exploited mainly due to high disease prevalence (Mengistu, Shimelis, Laing, & Lule, 2019) and soil acidity (Abebe, 2007).

As an important crop in several semiarid regions of the world, observed diversity in traits associated with tolerance to moisture stress suggest the potential of this collection to serve as an important source for development of resilient varieties adapted to diverse environments. Our results show large diversity for morphological traits (plant height, days to flowering and maturity), physiological traits (stay green and chlorophyll concentration) and agronomic traits such as grain yield, thousand kernel weight and seed number (Figure 2; Supplemental Table S4) that may have relations with drought tolerance. In an earlier study based on sorghum association panel, grain yield and yield components such as number of grains and grain weight per panicle were reported to be associated with drought tolerance (Mutava, Prasad, Tuinstra, Kofoid, & Yu, 2011). Likewise, a significant correlation (*P* ≤ .01) was observed between grain yield and yield components (seed number and thousand kernel weight) described in the current study (Supplemental Table S5). Stay‐green or delayed senescence in sorghum is an important trait associated with terminal drought tolerance and shows positive correlation with resistance to charcoal rot (Borrell, Mullet, George‐jaeggli, & Van Oosterom, 2014; Duncan, Bockholt, & Miller, 1981). Here, we report that a significant number of accessions displayed the stay green trait (Figure 2). SPAD is a chlorophyll meter used as alternative measurement tool for leaf greenness used for easy and quick assessments of genotype tolerance to moisture stress (Mutava et al., 2011; Silva, Jifon, Da Silva, & Sharma, 2007). In the current study, SPAD values were higher among materials from the dry areas compared with materials from all other agro‐climatic areas (Supplemental Table S4). Further correlation analysis among quantitative traits revealed positive association except for the negative correlation between SPAD with days to flowering, days to maturity, plant height and seed number. A similar study in barley indicated the negative correlation between days to heading and SPAD and positive correlation between grain yield and SPAD values (Bort, Araus, Hazzam, Grando, & Salvatore, 1998).

Ethiopian sorghum germplasm also harbors rare natural variants in the taxa. In this study, three double seeded genotypes were observed in the current SMIL collection (Figure 2). While their general impact on yield is not yet known, they clearly contribute to certain yield components such as number of kernels per panicle. A recent study identified ethyl methane sulfonate (EMS)‐induced *multiseeded (msd)* mutants that result in full spikelet fertility, and doubling grain number per panicle. And significance of doubled seeded genotypes was further demonstrated using a single base mutation which led to an increase in grain number by about 200% in sorghum (Gladman et al., 2019).

### 4.2 Genetic diversity and linkage disequilibrium

Molecular data demonstrated high diversity across the SMIL collection. A recent report on genomic characterization of a core subset of the Ethiopia sorghum within USDA‐NPGS, representing 17% of the world largest collection were described as diverse both phenotypically and genetically (Cuevas et al., 2017). Here we detected even higher diversity among the new SMIL collection than both USDA‐NPGS core subset and SAP. The SAP represented lower diversity and formed a distinct group. The uniqueness of the SAP from both Sudanese and Nigerien sorghum collection were similarly reported in recent studies (Cuevas & Prom, 2020; Maina et al., 2018).

The genome‐wide LD decay within 7–9 kb for the landrace collection for USDA‐NPGS and SMIL is in line with previous estimates, 7.3 kb at *r^2^  *= 0.2 among 3909 diverse sorghum landraces (Hu, Olatoye, Marla, & Morris, 2019). The LD decay to *r^2^*  = 0.2 was slightly faster in SAP which could be due to the recombination process through which the materials have undergone during the conversion program (Stephens et al., 1967).

### 4.3 Improved sorghum varieties from Ethiopia represent a narrow genetic diversity

PCA and unrooted neighbor‐joining tree, both revealed reduced genetic diversity across improved sorghum varieties including inbred lines in the pipeline and released landraces (Supplemental Figures S4 and S5). Moreover, almost half of the varieties had ancestry membership coefficient with likelihood of more than 0.90 (Figure 4c). This has revealed narrow genetic base among majority of the cultivars developed in the breeding program with two genotypes (Melkam/Meko/Misikir and Dagim/Birimash/IS9302) representing the genetic backgrounds of all cultivars. The improved cultivars Melkam, Meko and Misikir were introduced from ICRISAT, evaluated for local adaptation and eventually released for production in the dry lowlands of Ethiopia. The high ancestry (99%) shared between a commonly cultivated released landrace, Gambella1107, with Melkam/Meko/Misikir group suggests that Gambella1107 was likely used as parental source. Gambella1107 was selected from ‘zera zera’, a subgroup of the race caudatum, common in Ethiopian‐Sudan border (Rai, Murty, Andrews, & Bramel‐Cox, 1999). Most modern cultivars in Ethiopia are expected to have a background from the ‘zera zera’ sorghum landraces. In addition to low diversity, most of these varieties have been developed for dry lowland climate, also suggesting lack of breeding effort for the wet lowland areas. This observation implies that even in countries like Ethiopia that harbors wide genetic variability for the species, genetic uniformity arising from recycling of parental sources can be real. This explicitly can pose risks both to crop production due to genetic vulnerability and also drag progress in improvement due to lack of genetic variability. It is important that future cultivar improvement efforts utilize information from the current and other studies to maximize variability in the breeding parents while at the same time maintaining acceptable threshold of target traits.

### 4.4 Core collection

Sorghum core collections have been established across genebanks, particularly at ICRISAT and USDA‐NPGS where the largest number of landraces are being maintained. Subsets of reduced sizes have been selected following different sampling strategies and stratification of the entire collection based on agro‐morphological traits, response to photoperiod and passport data (Cuevas et al., 2017; Grenier & Hamon, 2001). However, the effect of environment on agro‐morphological traits and limitation of passport information, especially for old collections, hinders our ability to adequately capture the genetic diversity of an entire collection within the core subset. In the current study, the use of genotyping by sequencing coupled with agro‐morphological traits enabled the selection of a core collection representing the broader Ethiopian sorghum landrace germplasm.

Evaluation of diversity across accessions represented in the core subset through different methods (Supplemental Tables S9 to S11; Supplemental Figure S6) suggested maximum possible variation captured within 20% of the total collection. The newly developed core subset will, therefore, provide an easy access to efficient sorghum genetic resource utilization as the manageable size will enable easier multi‐location evaluation. The selected core will also provide guidance for further collection missions to acquire new accessions. The availability of molecular profile in addition to representative samples of the large germplasm is also important for selection of genetically diverse parents for trait introgression and making crosses to generate mapping populations for different traits of interest.

### 4.5 Genome‐environment association analysis revealed genetic basis of abiotic stress tolerance in sorghum

We have identified several loci and candidate genes underlying plant adaption to variable environments that could be causal or at LD with causal genes. Most of these genes were described to control adaptation to various abiotic factors such as cold, heat, salt and drought tolerance and protection against oxidative stress (Supplemental Table S14). Comparable genome‐wide association study on heat tolerance of sorghum developing leaves revealed several candidate genes associated to biological pathways involved in plant stress responses (Chen et al., 2017). Of the candidate genes suggested for various biological function, the *Srp40* and *Myb3* were implicated in plant developmental regulation (Ambawat et al., 2013; Califice et al., 2012). Our results also revealed two candidate genes, *F‐box* domain and *Srp40*, which were significantly associated to plant adaptation across environments with both annual mean temperature and altitude data. This may not be surprising as temperature is linearly associated with altitude (Figure 1d) and are considered complementary to each other. Our study also revealed a candidate gene, *Serine/Threonine‐protein kinase wnk with no lysine ‐related*, reported for possible roles in regulation of flowering time (Wang et al., 2008). The roles of environmental factors such as day length, temperature, drought and salinity in addition to endogenous genetic components determining flowering time across plant species including sorghum have been reported (Brunet & Larson‐rabin, 2012; Cho, Yoon, & An, 2017; Cuevas et al., 2016). It has also been possible to exploit potential of day length‐neutral sorghum genotypes of tropical origin that flower early in the temperate summer as it provides long days (Cuevas et al., 2016).

## 5 CONCLUSIONS

In this report, we demonstrated high diversity of the SMIL collection both in terms of phenotypic and genetic diversity parameters, population structure and LD. The faster LD decay within 9 kb across large and diverse landrace collection of sorghum will improve GWAS efficiency with high marker density. Narrow genetic diversity among the widely grown improved cultivars in Ethiopia, not representing the diversity in the landrace collections, displays underutilization of the existing genetic potential. This can also be considered risky due to increased recurrence of abiotic stresses and the changing pest and pathogen virulence as well as pathogen population dynamics. The genetic diversity and/or potential of these resources to grow across diverse environments has yet to be explored. Thus, it is recommended that breeders utilize diverse parental lines to capture novel genes or variants of existing genes/alleles for economic traits by taking advantage of the huge diversity in the Ethiopian sorghum germplasm. The developed core subset, representing the total diversity within SMIL collection, will provide easy access to germplasm by facilitating phenotyping across multiple environments, future genomics studies and, ultimately, selection of genotypes with desirable traits. Candidate loci identified with the GEA will have potential utilization for germplasm identification and sorghum breeding for stress prone area following further validation. The core collection needs further detailed characterization and cataloguing of additional traits for effective and comprehensive utilization in crop improvement.

## AUTHOR CONTRIBUTIONS

T.T., G.E. and T.M. conceived the project idea. T.B., A.S., A.N., C.B., M.M., A.G., D.L, K.D., G.A. and A.B. performed field experiments. G.G. analyzed data and wrote the paper. H.N., A.T., D.L., T.T., G. E. and T.M. revised the manuscript. T.M. supervised the study. All authors read and approved the final version of the manuscript.

## CONFLICT OF INTEREST DISCLOSURE

The authors declare no conflicts of interest.

## Supporting information

Table S1. Passport and phenotypic data of the SMIL collection, genetic cluster group and accessions included within core subset.

Table S2. List of morphological descriptors used to characterize sorghum germplasm collection.

Table S3. List of accessions from the US Sorghum Association panel (SAP) and USDA‐NPGS Ethiopian sorghum core subset used in this study

Table S4. Range and mean for seven quantitative traits across accessions originally collected from different sorghum growing agro‐ecologies of Ethiopia.

Table S5. Pearson correlation coefficients among seven quantitative descriptors (below diagonal) and confidence interval of the correlation coefficient at 95% (above diagonal).

Table S6. List of 1628 genotyped individuals from SMIL collection, intentional duplicates, respective genetic cluster group and duplicate accessions with distance below the established threshold value of 0.075.

Table S7. Ancestry fraction (Q) of accessions in the SMIL collection at different number of populations (K = 6, 8, 10 and 12).

Table S8. Ancestry fraction (Q) of improved lines and released landraces in Ethiopian sorghum breeding program.

Table S9. Shannon –Weaver diversity index (H') for categorical traits across entire and core sorghum landrace collections.

Table S10. Summary on frequency and Chi‐square (χ2) probability of entire and core sorghum accessions based on agro‐climatic information.

Table S11. Comparison of range, mean, and variances across entire and core sorghum landrace collections.

Table S12. Diversity statistics based on 106,330 SNP markers across 2198 sorghum accessions.

Table 13. Ancestry fraction (Q) among 2250 sorghum genotypes representing SMIL, NPGS and SAP Collection at K = 3 and K = 11.

Table S14. Significant single nucleotide polymorphisms (SNPs) and candidate genes across sorghum chromosome for agro‐climatic parameters.

Figure S1. Summary statistics on SNPs

Figure S2. Population genetic structure across SMIL collection.

Figure S3. Principal component analysis (PCA) showing sorghum accessions from different collection area categorized based on traditional climate classification.

Figure S4. Unrooted neighbor‐joining tree across SMIL collection.

Figure S5. PCA of 1553 genotyped accessions of landrace, released cultivars and inbred lines based on 72,190 SNP markers.

Figure S6. Unrooted neighbor‐joining tree for sorghum landrace including core subset selected based on 72,190 SNPs.

Figure S7. Genetic distance based on identity by state (IBS) among sorghum accessions based on 106,330 SNPs.

Figure S8. Distribution of the agro‐climatic data sets (mean annual precipitation, mean annual temperature and altitude) used for GWAS analysis.
