# Supplementary material for: A comprehensive phenotypic and genomic characterization of Ethiopian sorghum germplasm defines core collection and reveals rich genetic potential in adaptive traits
Source: Plant Genome. 2020 Sep 17;13(3):e20055. doi: 10.1002/tpg2.20055 (PMC12807287; doi:10.1002/tpg2.20055)
Supplement: Supplementary file 1 [file TPG2-13-e20055-s001.docx]

Table S2. List of morphological descriptors used to characterize sorghum germplasm collection.

| **Morphological traits** | **Description on data collected and parametric representation** |
| --- | --- |
| Days to flowering | Number of days from date of 50% emergence to date of 50% flowering |
| Physiological maturity | Number of days from date of 50% emergence of the field to date of physiological maturity |
| Plant height | Measurement in cm |
| SN | Seed number per panicle |
| GY | Grain yield per head (gm) |
| TGW | 1000 kernel weight (weight of 1000 kernels/seeds in grams at moisture content equal to or less than 12%) |
| SPAD | The Soil Plant Analysis Development chlorophyll meter reading value |
| Stay green | Scored with 1-5 scale at maturity where 1 = more green and 5 = less green |
| Leaf midrib colour | 1 = colorless (white), 2 = dull green, 3 = yellow and 4 = brown |
| Glume covering | Amount of kernel covered by glume recorded at maturity (Score: 1 = grain uncovered, 2 = 25% of grain covered, 3 = 50% of grain covered, 4 = grain fully covered, 6 = glumes longer than grain) |
| Awns | Presence and absence of awns (1 = awned and 2 = awnless) |
| Seed form | 1 = single and 2 = twin |
| Pericarp color | 1 = white, 2 = yellow, 3 = red, 4 = brown and 5 = buff |
| Kernel plumpness | 1 = dimple and 2 = plump |
| Panicle exsertion | 1 = well exserted more than 10cm between ligule of flag leaf to head base, 2 = 2-10cm exsertion, 3 = less than 2cm but ligule below the head base, 4 = peduncle recurved but head is below the ligule and clearly exposed splitting the leaf sheath, 5 = head covered by leaf sheath |
| Panicle compactness and shape | 1 = loose erect, 2 = loose drop, 3 = compact elliptic (erect) and  4 = compact oval or recurved |

GY = Grain yield per head (gm); TGW = thousand grain weight (gm); SN = seed number; PHT = plant height (cm); DTF = days to flowering; DTM = days to physiological maturity and SPAD = the Soil Plant Analysis Development chlorophyll meter.
