# Supplementary material for: A comprehensive phenotypic and genomic characterization of Ethiopian sorghum germplasm defines core collection and reveals rich genetic potential in adaptive traits
Source: Plant Genome. 2020 Sep 17;13(3):e20055. doi: 10.1002/tpg2.20055 (PMC12807287; doi:10.1002/tpg2.20055)
Supplement: Supplementary file 2 [file TPG2-13-e20055-s002.docx]

**Table S5.** Pearson correlation coefficients among seven quantitative descriptors (below diagonal) and confidence interval of the correlation coefficient at 95% (above diagonal).

| Observed traits | **DTF** | **DTM** | **PHT** | **TGW** | **GY** | **SN** | **SPAD** |
| --- | --- | --- | --- | --- | --- | --- | --- |
| DTF |  | [0.86,0.90] | [0.72,0.80] | [0.01,0.21] | [0.24,0.42] | [0.30,0.47] | [-0.59, -0.52] |
| DTM | 0.88^**^ |  | [0.62,0.73] | [0.08,0.27] | [0.15,0.34] | [0.21,0.39] | [-0.43, -0.35] |
| PHT | 0.77^**^ | 0.68^**^ |  | [0.01,0.20] | [0.25,0.43] | [0.29,0.47] | [-0.48, -0.41] |
| TGW | 0.11^*^ | 0.18^**^ | 0.10^*^ |  | [0.24,0.42] | [0.03,0.23] | [0.19,0.28] |
| GY | 0.33^**^ | 0.25^**^ | 0.35^**^ | 0.33^**^ |  | [0.76,0.83] | [-0.04,0.13] |
| SN | 0.38^**^ | 0.30^**^ | 0.38^**^ | 0.13^*^ | 0.79^**^ |  | [-0.08,0.01] |
| SPAD | -0.16^**^ | -0.39^**^ | -0.45^**^ | 0.24^**^ | 0.08^**^ | -0.03^NS^ |  |

*** P≤ 0.01, * P≤ 0.05, ^NS^ non-significant.*

*GY= Grain yield per head (gm); TGW = thousand grain weight (gm); SN = seed number; PHT = plant height (cm); DTF = days to flowering; DTM = days to physiological maturity and SPAD = the Soil* Plant *Analysis Development chlorophyll meter.*
