# Supplementary material for: A comprehensive phenotypic and genomic characterization of Ethiopian sorghum germplasm defines core collection and reveals rich genetic potential in adaptive traits
Source: Plant Genome. 2020 Sep 17;13(3):e20055. doi: 10.1002/tpg2.20055 (PMC12807287; doi:10.1002/tpg2.20055)
Supplement: Supplementary file 3 [file TPG2-13-e20055-s006.docx]

**Table S9.** Shannon –Weaver diversity index (H') for categorical traits across entire and core sorghum landrace collections.

| Traits | H' | |
| --- | --- | --- |
|  | EC | CC |
| Seed form | 0.15 | 0.17 |
| Leaf mid rib colour | 0.56 | 0.56 |
| Stay green | 0.55 | 0.55 |
| Head exertion | 0.59 | 0.57 |
| Head compactness and shape | 0.62 | 0.61 |
| Awns | 0.55 | 0.55 |
| Kernel covering | 0.48 | 0.49 |
| Kernel colour | 0.63 | 0.63 |
| Kernel plumpness | 0.24 | 0.24 |

*EC = Entire collection consisting of 1941 lines; CC = Core collection consisting of 387 lines*
