# Supplementary material for: A comprehensive phenotypic and genomic characterization of Ethiopian sorghum germplasm defines core collection and reveals rich genetic potential in adaptive traits
Source: Plant Genome. 2020 Sep 17;13(3):e20055. doi: 10.1002/tpg2.20055 (PMC12807287; doi:10.1002/tpg2.20055)
Supplement: Supplementary file 4 [file TPG2-13-e20055-s007.docx]

**Table S10.** Summary on frequency and Chi-square (χ2) probability of entire and core sorghum accessions based on agro-climatic information.

| Climate classification | EC | CC | df | χ2 | *P* |
| --- | --- | --- | --- | --- | --- |
| Across all climate | 1941 | 387 | 6 | 6.87 | 0.44 |
| Dry highland | 135 | 36 | 1 | 2.42 | 0.12 |
| Dry intermediate altitude | 132 | 23 | 1 | 0.36 | 0.55 |
| Dry lowland | 346 | 57 | 1 | 1.41 | 0.24 |
| Wet highland | 668 | 135 | 1 | 0.02 | 0.89 |
| Wet intermediate altitude | 211 | 35 | 1 | 1.02 | 0.31 |
| Wet lowland | 376 | 84 | 1 | 0.89 | 0.35 |
| Unknown | 73 | 17 | 1 | 0.33 | 0.56 |

*EC = Entire collection; CC= Core collection; df = degrees of freedom*
