# Supplementary material for: A comprehensive phenotypic and genomic characterization of Ethiopian sorghum germplasm defines core collection and reveals rich genetic potential in adaptive traits
Source: Plant Genome. 2020 Sep 17;13(3):e20055. doi: 10.1002/tpg2.20055 (PMC12807287; doi:10.1002/tpg2.20055)
Supplement: Supplementary file 5 [file TPG2-13-e20055-s008.docx]

**Table S11.** Comparison of range, mean, and variances across entire and core sorghum landrace collections.

| **Trait** | **Range** | | **Mean** | | **Variance** | |  | |
| --- | --- | --- | --- | --- | --- | --- | --- | --- |
|  | **EC** | **CC** | **EC** | **CC** | **EC** | **CC** | ***F* value** | ***P*** |
| Days to 50% flowering | 64.00 – 233.80 | 66.00 – 216.00 | 111.47^a^ | 110.93 ^a^ | 422.38 | 436.04 | 0.97 | 0.16 |
| Days to maturity | 110.50 – 288.00 | 110.50 – 270.00 | 165.45 ^a^ | 165.54 ^a^ | 463.73 | 478.92 | 0.97 | 0.18 |
| Plant height (cm) | 65.00 – 586.67 | 78.00 – 567.00 | 309.66 ^a^ | 305.75 ^a^ | 6,841.69 | 7181.64 | 0.95 | 0.07 |
| 1000 kernel weight | 2.90 – 77.00 | 2.90 – 72.00 | 25.22 ^a^ | 24.63 ^a^ | 58.89 | 57.18 | 1.03 | 0.28 |
| Grain yield (g) | 0.10 – 864.40 | 0.40 – 748.00 | 149.04 ^a^ | 140.82 ^a^ | 20,564.70 | 19,766.29 | 1.04 | 0.26 |
| Seed number per panicle | 20.00 – 8070.59 | 40.00 –7333.33 | 2,314.30 ^a^ | 2,207.38^a^ | 1,482,313.44 | 1,296,351.86 | 1.14 | 0.06 |
| SPAD | 19.70 – 69.76 | 30.30 - 68.68 | 49.11 ^a^ | 48.90 ^a^ | 46.97 | 51.86 | 0.91 | 0.10 |

Means followed by the same letter are not significantly different at *P = 0.05*

*EC = Entire collection; CC = Core collection*
