# Supplementary material for: A comprehensive phenotypic and genomic characterization of Ethiopian sorghum germplasm defines core collection and reveals rich genetic potential in adaptive traits
Source: Plant Genome. 2020 Sep 17;13(3):e20055. doi: 10.1002/tpg2.20055 (PMC12807287; doi:10.1002/tpg2.20055)
Supplement: Supplementary file 6 [file TPG2-13-e20055-s009.docx]

**Table S12.** Diversity statistics based on 106,330 SNP markers across 2,198 sorghum accessions.

| Per sample statistics | | | | |
| --- | --- | --- | --- | --- |
|  | | NPGS | SAP | SMIL |
| F_IS_ | Min. | 0.0785 | 0.3750 | 0.0131 |
|  | Median | 0.7142 | 0.7918 | 0.7230 |
|  | Mean | 0.6824 | 0.7844 | 0.6390 |
|  | Max. | 0.8923 | 0.9115 | 0.8925 |
|  | SD | 0.1369 | 0.0653 | 0.2056 |
| H | Min. | 0.0282 | 0.2297 | 0.0284 |
|  | Median | 0.0762 | 0.0562 | 0.0764 |
|  | Mean | 0.0836 | 0.0579 | 0.0972 |
|  | Max. | 0.3640 | 0.1659 | 0.3491 |
|  | SD | 0.0357 | 0.0174 | 0.0527 |
| Per marker statistics | | | | |
| MAF | Min. | 0.0000 | 0.0000 | 0.0000 |
|  | Median | 0.1766 | 0.1687 | 0.1812 |
|  | Mean | 0.2043 | 0.1942 | 0.2065 |
|  | Max. | 0.5000 | 0.5000 | 0.5000 |
|  | SD | 0.1394 | 0.1495 | 0.1389 |
| Ho | Min. | 0.0000 | 0.0000 | 0.0000 |
|  | Median | 0.0363 | 0.0103 | 0.0527 |
|  | Mean | 0.0928 | 0.0581 | 0.1051 |
|  | Max. | 0.9838 | 1.0000 | 0.9975 |
|  | SD | 0.1458 | 0.1232 | 0.1516 |
| He | Min. | 0.0000 | 0.0000 | 0.0000 |
|  | Median | 0.2949 | 0.2921 | 0.3030 |
|  | Mean | 0.2890 | 0.2768 | 0.2942 |
|  | Max. | 0.5000 | 0.5000 | 0.5000 |
|  | SD | 0.1494 | 0.1651 | 0.1473 |
| PIC | Min. | 0.0000 | 0.0000 | 0.0000 |
|  | Median | 0.2489 | 0.2392 | 0.2527 |
|  | Mean | 0.2343 | 0.2162 | 0.2361 |
|  | Max. | 0.3750 | 0.3750 | 0.3750 |
|  | SD | 0.1087 | 0.1273 | 0.1092 |

F_IS_ = Inbreeding coefficient; H = Proportion heterozygous; MAF = minor allele frequency; Ho = Observed heterozygosity; He = expected heterozygosity; PIC = polymorphic information content; SD = standard deviation
