# Supplementary material for: A comprehensive phenotypic and genomic characterization of Ethiopian sorghum germplasm defines core collection and reveals rich genetic potential in adaptive traits
Source: Plant Genome. 2020 Sep 17;13(3):e20055. doi: 10.1002/tpg2.20055 (PMC12807287; doi:10.1002/tpg2.20055)
Supplement: Supplementary file 7 [file TPG2-13-e20055-s022.docx]

**Table S4.** Range and mean for seven quantitative traits across accessions originally collected from different sorghum growing agro-ecologies of Ethiopia.

| **Trait** | **Summary Statistics** | **Sorghum growing agro-ecologies** | | | | | | | |
| --- | --- | --- | --- | --- | --- | --- | --- | --- | --- |
|  |  | **Dry lowland (n=382)** | **Wet lowland (n=378)** | **Dry intermediate altitude (n=134)** | **Wet intermediate altitude (n=226)** | **Dry highland (n=135)** | **Wet highland (n=682)** | **Unknown (n=73)** | **Overall (n=2010)** |
| DTF | Range | 75.70-147.63 | 76.32-154.52 | 80.67 -147.50 | 79.10-150.50 | 77.50-146.84 | 79.07-157.25 | 79.37-149.61 | 75.70-157.25 |
|  | Mean ± SE | 101.79 ± 0.72 | 115.04 ± 1.01 | 108.05 ± 1.11 | 120.63 ± 1.14 | 106.37 ± 1.14 | 112.77 ± 0.59 | 112.15 ± 2.03 | 111.23 ± 0.38 |
| DTM | Range | 136.25-205.00 | 140.20-207.60 | 144.80-192.8 | 143.2-203.00 | 142.64-197 | 140.00-208.5 | 140.00-202.6 | 136.25-208.5 |
|  | Mean ± SE | 158.80 ± 0.58 | 169.32 ± 0.81 | 161.93 ± 0.84 | 173.05 ± 0.9 | 160.80 ± 0.90 | 165.99±0.49 | 166.22 ± 1.70 | 165.44 ± 0.31 |
| PHT | Range | 108.94-470.27 | 126.00-455.83 | 177.22-421.3 | 149.50-482.3 | 131.49-460.6 | 114.33-490.6 | 142.17-442.5 | 108.94-490.7 |
|  | Mean ± SE | 272.63 ± 3.87 | 303.06 ± 3.54 | 303.21 ± 4.63 | 331.33 ± 4.16 | 302.18 ± 5.76 | 322.56 ± .22 | 317.49 ± 7.78 | 307.54 ± .51 |
| TGW | Range | 11.57 – 44.80 | 7.50 - 42.80 | 13.00- 38.50 | 7.00-40.50 | 11.50 - 39.50 | 7.00 - 52.00 | 13.20-41.03 | 7.00 - 52.00 |
|  | Mean ± SE | 26.92 ± 0.38 | 23.73 ± 0.30 | 26.53 ± 0.46 | 23.27 ± 0.46 | 25.19 ± 0.47 | 25.57 ± 0.27 | 24.59 ± 0.81 | 25.23 ± 0.69 |
| GY | Range | 1.10 - 185.8 | 0.10 -173.30 | 2.90 – 197.80 | 1.30 -190.4 | 0.70 - 212.70 | 0.30 - 201.60 | 0.80 -123.70 | 0.10 - 212.70 |
|  | Mean ± SE | 54.45 ± 1.48 | 51.36 ± 1.40 | 62.49 ± 2.92 | 58.63 ± 2.19 | 55.19 ± 3.24 | 60.01 ± 1.21 | 56.15 ± 2.88 | 56.92 ± 0.70 |
| SN | Range | 55.00-6466.67 | 142.86-6718.75 | 258.06-6181.3 | 104.00-7333.3 | 58.33-6591.3 | 20.00-8070.6 | 137.50-6221.4 | 20.00-8070.6 |
|  | Mean ± SE | 2023.29 ±56.9 | 2200.75±59.62 | 2320.54±101.6 | 2600 ± 82.12 | 2157.86±107.1 | 2447.67±47.9 | 2480.30±143.8 | 2309.23±27.1 |
| SPAD | Range | 36.04-68.68 | 26.70-63.38 | 19.70-66.36 | 35.20-69.76 | 30.30 - 62.70 | 25.12-66.62 | 34.94-60.96 | 19.70-69.76 |
|  | Mean ± SE | 51.74 ± 0.34 | 48.14 ± 0.37 | 50.06 ± 0.64 | 48.31±0.42 | 49.73 ± 0.59 | 48.40 ± 0.24 | 48.70 ± 0.80 | 49.20 ± 0.15 |

*GY= Grain yield per head (gm); TGW = thousand grain weight (gm); SN = seed number; PHT = plant height (cm); DTF = days to flowering; DTM = days to physiological maturity and SPAD = the Soil* Plant *Analysis Development chlorophyll meter.*
